# Visual Illusions: An Interesting Tool to Investigate Developmental Dyslexia and Autism Spectrum Disorder

**DOI:** 10.3389/fnhum.2016.00175

**Published:** 2016-04-25

**Authors:** Simone Gori, Massimo Molteni, Andrea Facoetti

**Affiliations:** ^1^Department of Human and Social Sciences, University of BergamoBergamo, Italy; ^2^Child Psychopathology Unit, Scientific Institute, IRCCS Eugenio MedeaBosisio Parini, Italy; ^3^Developmental and Cognitive Neuroscience Lab, Department of General Psychology, University of PadovaPadua, Italy

**Keywords:** illusory effect, perception, attention, autistic traits, reading disorder

## Abstract

A visual illusion refers to a percept that is different in some aspect from the physical stimulus. Illusions are a powerful non-invasive tool for understanding the neurobiology of vision, telling us, indirectly, how the brain processes visual stimuli. There are some neurodevelopmental disorders characterized by visual deficits. Surprisingly, just a few studies investigated illusory perception in clinical populations. Our aim is to review the literature supporting a possible role for visual illusions in helping us understand the visual deficits in developmental dyslexia and autism spectrum disorder. Future studies could develop new tools – based on visual illusions – to identify an early risk for neurodevelopmental disorders.

## Visual Illusion as a Tool to Investigate Brain Processing

A visual illusion refers to a percept that is different from what would be typically predicted based on the physical stimulus. Illusory perception is often experienced as a real percept. Illusory motion should not be confused with the perception of dynamism evoked by paintings (Gori et al., 2008b), or by the speed lines often used in comic books (Burr, 2000). On the contrary, the observer’s percept is usually phenomenally indistinguishable from real motion (see Kitaoka and Ashida, 2007; Gori and Stubbs, 2014, for a review on motion illusions).

Visual illusions reflect the constraints developed by our visual system, through evolution, to support the efficient formation of visual representations that are also sufficient in describing our external environment (see Eagleman, 2001, for a review). For example, such constraints allow us to perceive the constant illusion of a stable perceptual world, despite our receptors continuously signaling motion due to eye, head, and body movements. Consequently, it is quite difficult to provide a rigorous definition of “visual illusion”, since our visual experiences are, in effect, always an illusion (Eagleman, 2001; Rogers, 2014). As nicely described by Spillmann (2009), visual illusions show “the brain’s signature superimposed upon the stimulus”. Thus, visual illusions provide a powerful, non-invasive means by which to investigate our brain mechanisms.

There are numerous types of visual illusions, and they can roughly categorized by their perceptual appearance, including brightness (e.g., Pinna et al., 2003; Gori and Stubbs, 2006; Todorović, 2006; Anstis et al., 2007; Spillmann et al., 2010; Galmonte et al., 2015), color (e.g., Varin, 1971; Pinna et al., 2001), size (e.g., Sperandio et al., 2010, 2012, 2013; Sperandio and Chouinard, 2015), etc. (see Gregory, 1968, 1997). However, illusion classification did not reach a general consensus yet (see Hamburger, 2016, for a recent review). Measuring a visual illusion, even if it sounds counterintuitive, can be done in a very accurate way in both human and non-human species (e.g., Macknik and Livingstone, 1998; Conway et al., 2005; Gori et al., 2006, 2008a, 2010a,b, 2014; Fantoni and Pinna, 2008; Giora and Gori, 2010; Gori and Spillmann, 2010; Spillmann et al., 2010; Hamburger, 2012; Ito, 2012; Otero-Millan et al., 2012; Shi et al., 2013; Agrillo et al., 2015). Following the everyman’s perspective, visual illusions could not be considered a good tool for investigating perceptual differences in groups because they are “subjective”. “Subjective” would mean that the observer response would be based on her/his perceptual outcome and also on her/his response criterion. On the contrary, following this naive interpretation, tasks based on physical differences in the stimuli would be “objective”. This distinction is based on the wrong assumption so-called, “naive realism”, which implies that perception faithfully reflects the stimulus at the retina level (Spillmann, 2009). Such an approach fails to consider the fact that performance in behavioral tasks reflect processing well-beyond that of our low-level visual systems, e.g., retinal processes. Consequently, the supposed “objective” task will be subjective exactly like a “subjective” task, because they are both a result of a mixture of perceptual processing and response criteria. As a side note, any motion stimulus presented on a computer screen is illusory motion exactly like cinema is. Thus, almost all the motion studies in the last 30 years are based on illusory motion instead of real motion.

In summary, the use of visual illusions to investigate brain processing and perceptual differences among groups in behavioral studies of perception is legit exactly like employing ordinary stimuli. Moreover, measures of visual illusions can provide much more information about neural mechanisms than ordinary stimuli due to their ability to highlight the visual system constraints. Over the course of vision science history, several illusions successfully provided the first intuition of how the brain processes a stimulus or the tool to investigate the neurobiological characteristics of the visual system (see Eagleman, 2001, for a review).

Although the study of clinical populations and the world of visual illusions may not seem very connected, there have been some excellent advancements resulting from their joint study. For example, Notredame et al. (2014) recently reviewed the contribution of visual illusions in understanding schizophrenia. Here, we aim to review the literature using visual illusions to understand two of the most studied neurodevelopmental disorders: developmental dyslexia (DD) and autism spectrum disorder (ASD). Our choice of DD and ASD primarily stems from reports of peculiar visual abilities characterizing these clinical populations. Consequently, they seems perfect candidate disorders for investigation by visual illusions. Relevant articles for this review were obtained through a keyword search of the PubMed database, with dyslexia AND visual illusions” and “autism AND visual illusions” as search terms.

In addition to our review, we provide ideas for potential new horizons and the advancement of DD and ASD research through the study of visual illusions.

## Visual Deficits in Developmental Dyslexia

DD is often defined as a deficit in reading acquisition despite normal intelligence and access to conventional instruction (e.g., American Psychiatric Association Task Force on DSM-IV, 1994). However, some variations in the definition of DD are present. For example, the U.S. National Institutes of Health define DD as a learning disorder (NINDS, 2001/2015). One of the worldwide used manual of medical diagnosis ICD 10 (1992) includes separate diagnoses for DD and for “dyslexia and alexia”. The latest version of the U.S. manual of psychiatric diagnosis (American Psychiatric Association Task Force on DSM-V, 2013), does not specifically define DD including that in a larger category called specific learning disorders.

Several attempted to account for DD, two main views, however, received major support. The first approach proposes that DD arises from deficits in systems that are specifically linguistic in nature. In particular, the phonological deficit theory suggests that DD arises from deficits in phonological processing (e.g., Goswami, 2003). In contrast, many authors suggest that disorders in underlying non-linguistic sensory mechanisms are the real core deficit in DD (e.g., Stein and Walsh, 1997; Vidyasagar and Pammer, 2010; Facoetti, 2012; Gori and Facoetti, 2014, 2015; for visual deficits; Wright et al., 2000; Tallal, 2004, for auditory deficits).

This theory, known as the temporal processing hypothesis, is the multisensory (i.e., visual and auditory) version of the magnocellular dorsal (M-D) theory of DD. This theory suggests that children with DD have deficits in rapid processing in visual and auditory modalities (see Farmer and Klein, 1995; Hari and Renvall, 2001, for reviews). Chiefly, the temporal processing hypothesis explicitly claims that phonological decoding (i.e., pseudo word reading) deficits in dyslexics could arise from impairments in sensory processing of visual and auditory dynamic-stimuli (e.g., Geiger et al., 2008; Lallier et al., 2010; Facoetti et al., 2010a,b; Harrar et al., 2014).

The well-known M-D theory of DD is often referred specifically to the visual modality, and it is a comprehensive, albeit controversial account (e.g., Amitay et al., 2002; Sperling et al., 2005, 2006; Olulade et al., 2013). This theory stems from the observation that reading disabled individuals are impaired in the specific visual M-D pathway (e.g., Livingstone et al., 1991; see Stein and Walsh, 1997; Boden and Giaschi, 2007; Vidyasagar and Pammer, 2010; Vidyasagar, 2013; Gori and Facoetti, 2014, 2015; Pammer, 2014, for reviews).

The M-D pathway originates in the ganglion cells of the retina, passes through the M-layer of the lateral geniculate nucleus (LGN), and finally reaches the occipital and parietal cortices (Maunsell and Newsome, 1987). The M-D stream is considered blind to colors, and responds optimally to contrast differences, low spatial frequencies, high temporal frequencies, and motion (Livingstone and Hubel, 1987). The M-D stream mediates the required sensorimotor transformations for visually guided actions (see Goodale and Milner, 1992, for a review).

The M-D stream seems to be impaired in individuals with DD, whereas the other major parallel pathway of the visual system, the parvocellular-ventral (P-V) stream, is intact (Stein and Walsh, 1997; Stein, 2001; Boden and Giaschi, 2007; Vidyasagar and Pammer, 2010).

The P-V pathway is characterized by both lower temporal resolution and superior sensitivity to high spatial frequencies, and it is also sensitive to color changes (Livingstone and Hubel, 1987). The P-V stream plays its major role in the perceptual identification of objects (Goodale and Milner, 1992). However, some studies failed to confirm differences in high temporal, low spatial frequency stimulus perceptions, which are thought to rely upon M-D processing, between individuals with DD and controls (e.g., Victor et al., 1993; Johannes et al., 1996; Williams et al., 2003; see Schulte-Körne and Bruder, 2010, for a review).

Importantly, a post mortem study showed that in the brains of individuals with DD the M neurons of the LGN were noticeably smaller than those found in normal readers’ brains, while the P neurons did not differ (Livingstone et al., 1991). This finding was recently buttressed by the first *in vivo* MRI study (Giraldo-Chica et al., 2015), showing smaller LGN volume in a larger sample of individuals with DD compared to controls.

Gori et al. (2015a) also found the first reported association between a genetic variance (the DCDC2-Intron 2 deletion) and an M-D deficit in both individuals with DD and typical readers. The DCDC2-Intron 2 deletion is a proved DD genetic risk factor (e.g., Meng et al., 2005; Mascheretti et al., 2013; Marino et al., 2014; Riva et al., 2014).

Recently, Gori et al. (2015b) showed that: (i) motion perception was impaired in children with dyslexia in comparison both to age matched and to reading level (RL) controls, that are younger controls who read at the same level as the dyslexics (Goswami, 2003); (ii) pre-reading visual motion perception—independently from auditory-phonological skill—predicted future reading development, and; (iii) targeted M-D trainings—not involving any auditory-phonological stimulation—led to improved reading skill in children and adults with DD. Their findings demonstrate, for the first time, a causal relationship between M-D deficits and DD, virtually closing a 30 years long debate.

It should be noted that the M-D pathway terminates mainly in the posterior parietal cortex, which is highly linked to the prefrontal regions (Mishkin and Ungerleider, 1982; Goodale and Milner, 1992; Merigan and Maunsell, 1993). These cortical regions rapidly control selective attention in humans (e.g., Facoetti and Molteni, 2000; Koyama et al., 2013; Ronconi et al., 2014a, 2016; see Corbetta and Shulman, 2002, 2011; Dosenbach et al., 2008, for reviews). Thus, a weakened or abnormal M-D input to the dorsal-stream could result in a spatial and temporal attention deficit in children and adults with DD (e.g., Brannan and Williams, 1987; Williams et al., 1987; Valdois et al., 1995; Hari et al., 1999; Vidyasagar and Pammer, 1999; Facoetti et al., 2000, 2001, 2005, 2008, 2010b; Iles et al., 2000; Hari et al., 2001; Bosse et al., 2007; Buchholz and Aimola Davies, 2007; Lobier et al., 2014; see Vidyasagar, 1999; Hari and Renvall, 2001; Boden and Giaschi, 2007; Vidyasagar and Pammer, 2010; Stein, 2014, for reviews) and specifically in dyslexics with poor pseudo-word reading ability (Cestnick and Coltheart, 1999; Buchholz and McKone, 2004; Facoetti et al., 2006, 2010a; Roach and Hogben, 2007; Jones et al., 2008; Ruffino et al., 2010, 2014). Interestingly, the M-D and attentional deficits in individuals with DD were found also in logographic languages such as Chinese (e.g., Zhao et al., 2014; Liu et al., 2015). The visual attentional disorder is now recognized as a core deficit of DD (Franceschini et al., 2012, 2013; Zorzi et al., 2012; see Stein, 2014; Franceschini et al., 2015, for reviews).

## Visual Illusions in Developmental Dyslexia

The role of visual illusions in unveiling perceptual and attentional deficits in DD was crucial during the last 15 years. For example, Slaghuis et al. (1996) using an apparent motion Ternus display (Ternus, 1938) showed that individuals with DD, both children and adults, presented a significant reduction in Ternus group movement.

Ternus (1938, see **Table 1** for details) devised an apparent motion stimulus composed of two alternating frames separated by a blank interframe interval (e.g., Alais and Lorenceau, 2002).

**Table 1:** Descriptions of the visual illusions used in the studies reviewed in this article.

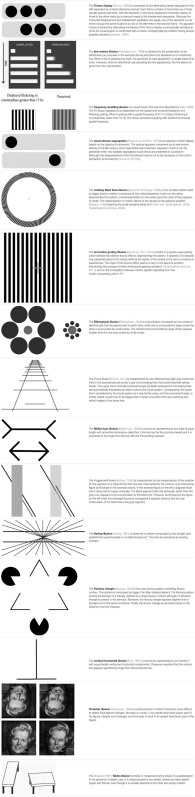

The reduced group motion perception in individuals with DD was interpreted as an increase in the duration of visible persistence (Slaghuis et al., 1996), which was consistent with evidence for an M-D pathway disorder in DD (e.g., Livingstone et al., 1991).

Slaghuis and Ryan (1999) also using the Ternus display confirmed the results by Slaghuis et al. (1996) and gave the same explanation in terms of increased visible persistence in children with DD.

Cestnick and Coltheart (1999), employed the Ternus display as well, showing differences between individuals with DD and controls. Their results indicate that the Ternus task performance was related to pseudoword reading but not to exception word reading ability (i.e., phonological DD subtype). These authors provided two alternative interpretations of these findings: (1) pseudoword reading requires a serial left-to-right allocation of visual attention across the letter string being read. The neural systems involved in this attentional process also play a part in responses to the Ternus display; or (2) poor pseudoword reading and abnormal Ternus performance were not directly related: perinatal/neurodevelopmental insult affected the LGN (influencing Ternus performance) and the adjacent medial geniculate nucleus (affecting phonological ability), and the medial geniculate nucleus abnormalities may be more functionally related to poor non-word reading. Davis et al. (2001) confirmed the results found by Cestnick and Coltheart (1999) demonstrating that individuals with DD do genuinely differ from typical readers in their perceptual processing.

Pammer and Lovegrove (2001) convincingly suggested the Ternus display as a proxy able to test the M pathway functioning in DD. Jones et al. (2008) failed in finding significant differences in the group motion using Ternus display between high functioning adults with DD and a control group. The authors themselves provide one explanation for these results. The M deficit in their specific group of university students, with a high degree of compensation, is so mild that difficulty only arises when M input is required for the direction of sequential attention (Vidyasagar, 1999, 2013; Facoetti, 2012). Moreover, in the Jones et al. (2008) study, the group with DD was unselected and no subtyping was done. Cestnick and Coltheart (1999) found differences in the Ternus display performance specifically in the phonological DD subtype.

Pammer and Wheatley (2001) showed that individuals with DD are less sensitive to the detection of the frequency doubling (FD) illusion pattern than normal readers, supporting a low-level deficit in the M-D pathway. The FD illusion (see **Table 1** for details) was explained by Kelly (1981) in terms of the full wave rectification carried out by the visual system. Such rectification is found in M(y)-cells of the primate retina (Benardete et al., 1992) and LGN (Kaplan and Shapley, 1982; Marrocco et al., 1982). It is therefore suggested, that responses from the M(y)-cells underlie perception of the FD illusion (see Maddess et al., 1992, for a detailed discussion regarding the relationship between M(y)-cells and frequency doubling).

Buchholz and McKone (2004) confirmed the results of Pammer and Wheatley (2001) showing deficits in both M pathway (FD illusion) and attentional tasks. These deficits cannot be explained by differences in concentration lapses, eye movements or reaction times between individuals with DD and controls.

Pammer et al. (2004) showed that a poor performance in the FD illusion task was associated with a lower ability to read a word inside the text, which could suggest a dysfunctional attentional focus in individuals with DD.

Kevan and Pammer (2008) demonstrated that children at familial risk for DD already show a higher threshold for the FD illusion even at pre-reading stage. Importantly, in their longitudinal study (Kevan and Pammer, 2009), the threshold for the FD illusion at the pre-reading stage predicts future reading skills, suggesting a causal role of the M-D deficit in DD.

Gori et al. (2014) recently demonstrated that children with DD showed a lower performance in the FD illusion task not only in comparison with an age matched control group, but also with an RL control group. The use of the RL control can be a first step in research aimed at delineating the causal factors in reading disabilities.

In addition, Steinman et al. (1998) employed the line-motion illusion (Hikosaka et al., 1993) to show how the spatial distribution of attention (e.g., Facoetti and Molteni, 2001) – which is dominated by the M input – differs between individuals with DD and typical readers.

The line motion illusion is obtained by the presentation of an attentional cue just before the appearance of a stationary line (see **Table 1** for details). This stimulus revealed the connection between the M-D pathway and the attentional deficit in DD (Steinman et al., 1998). Recently, Hamm et al. (2014) showed, using fMRI, that the pattern of brain activation during a motion direction task while observing the line motion illusion was very consistent with the attentional gradient model. These results confirmed that this stimulus is a good proxy for measuring the attentional abilities of individuals with DD.

Lallier et al. (2009) employed an apparent motion display coined by Bregman and Achim (1973) the “visual stream segregation”. The “visual stream segregation” (Bregman and Achim, 1973) is an apparent motion display based on the classical β movement (see **Table 1** for details). The illusion persists even when the stimuli are widely separated, a phenomenon called “long-range apparent motion” (Braddick, 1980; Maloney et al., 2005; Schiller and Carvey, 2006). In order to respond to apparent motion, neurons have to integrate information over a large part of visual space, spanning at least the distance between the two inducing stimuli. Several studies have shown that MT in the macaque and other primates and its human homolog, the MT/V5 complex (hMT/V5+), respond to stimulus conditions that induce apparent motion (e.g., Mikami, 1991). In contrast, receptive-field sizes in early visual areas and, in particular, the primary visual cortex (V1) are too small to account for long-range interactions between stimuli. The fact that one actually observes spatially resolved movement between the inducing stimuli in apparent motion suggests that there could be a filling-in process in early visual areas that is driven by feedback from extrastriate regions with larger receptive-field sizes. In particular, back-projections from hMT/V5+ have been shown to be relevant for perception of motion and apparent motion (e.g., Pascual-Leone and Walsh, 2001). Muckli et al. (2005) proved that the activity in V1 changes as a function of the subjectively perceived motion trace. Sterzer et al. (2006) showed, using fMRI, how the hMT/V5+ feedback mediated the activation of V1 in the presence of apparent motion.

The visual stream segregation was used in conjunction with an auditory stream segregation to assess the “sluggish attentional shifting” (SAS) hypothesis (Lallier et al., 2009) suggested by Hari and Renvall (2001). This theory argues that individuals with DD would show attentional deficit, through SAS, in all sensory modalities. The amodality assumption of the SAS theory was never directly assessed before in the same group of individuals with DD using similar paradigms in both the visual and auditory modalities. Lallier et al. (2009) showed a deficit in both auditory and visual modality in individuals with DD. These results supported the view that the multisensory SAS hypothesis. Lallier et al. (2010) confirmed these results adding also evoked related potentials (ERPs) evidence.

Gori et al. (2015a) showed that children with DD presented a lower performance also in tasks specifically tapping the D portion of the M-D pathway in comparison with both age controls and RL controls. Moreover, Gori et al. (2015a) reported, for the first time, an association between a genetic variance (a deletion in intron 2 of the DCDC2 gene), and an M-D deficit in individuals with and without DD. In order to obtain these results, two motion illusions were employed: the rotating tilted lines illusion (RTLI) (Gori and Hamburger, 2006) and the accordion grating illusion (AGI) (Gori et al., 2011, 2013).

The RTLI, is the simplest pattern able to trigger illusory rotation in the presence of only radial expansion motion on the retina (see **Table 1** for details). The illusory effect appears to be strongly reduced or even to disappear with isoluminant colors (Hamburger, 2012).

The second motion illusion employed by Gori et al. (2015a) was the AGI consisting in a square-wave grating which exhibits two distinct illusory effects (see **Table 1** for details). The V5/MT, which is a core, neural station of the M-D pathway, also processes the illusory motion perception (Ruzzoli et al., 2011). Consequently, the RTLI and the AGI represent appropriate candidates for testing the functioning of this visual pathway. The choice of testing illusory motion was not only interesting for itself, but it also provided some advantages in comparison to testing the real motion perception. The main technical advantage was that the illusory motion required more contrast than real motion to be perceived, which provided a larger window to vary the independent variable. The direct consequence was having a more sensitive instrument to test the M-D pathway functionality. Moreover, most of the evidence for the visual D deficit in DD has derived from studies of coherent dot motion perception (see Stein, 2014, for a recent review). However, the impairment in the coherent dot motion task was only found in the presence of high external noise (Sperling et al., 2006). The illusory motion tasks were the first able to measure the more dorsal portion of the M-D pathway being clearly independent from any noise exclusion mechanism. The RTLI was also used to show the M-D functioning improvement after action video game training in children with DD (Gori et al., 2015b).

Finally, although it is out of the scope of this review on visual illusions, it is interesting to mention that auditory illusions also played an important role in the study of DD (e.g., Hari and Kiesila, 1996; Helenius et al., 1999; Lallier et al., 2009, 2010).

In summary, visual illusions played a crucial role in the study of DD, and it is probably only the starting point of a successful long collaboration between scientists studying this neurodevelopmental disorder and researchers in vision sciences. In particular, the above described visual illusions clearly played a prominent role in providing important evidence for the M-D theory and the attentional deficits in DD. Interestingly, the results obtained by the reviewed visual illusions not only helped in showing an association between perceptual and attentional deficits and DD, but also provided often the first evidence of a causal role of these cognitive deficits on the emergence of DD (see **Table 2** for a summary of reviewed studies).

**Table 2:** Overview of the published articles investigating visual illusions sensitivity in developmental dyslexia (DD).

Reference	Visual Illusion	Main results (i.e., illusion sensitivity)
Slaghuis et al., 1996	Apparent motion Ternus display	DD < Controls in perceived group motion
Steinman et al., 1998	Line-motion illusion	DD < Controls
Slaghuis and Ryan, 1999	Apparent motion Ternus display	DD (only mixed subtype of dyslexia) < Controls in perceived group motion
Cestnick and Coltheart, 1999	Apparent motion Ternus display	DD (only phonological subtype of dyslexia) < Controls in perceived group motion
Davis et al., 2001	Apparent motion Ternus display	DD < Controls n perceived group motion
Pammer and Wheatley, 2001	Frequency doubling illusion	DD < Controls
Buchholz and McKone, 2004	Frequency doubling illusion	DD < Controls
Jones et al., 2008	Apparent motion Ternus display	DD = Controls
Kevan and Pammer, 2008	Frequency doubling illusion	Pre-readers at familial risk for DD < Pre-readers not at risk
Kevan and Pammer, 2009	Frequency doubling illusion	Future poor readers < Future typical readers
Lallier et al., 2009	Visual stream segregation	DD < Controls
Lallier et al., 2010	Visual stream segregation	DD < Controls
Gori et al., 2014	Frequency doubling illusion	DD (only children) < in comparison to both chronological-age and reading-level Controls
Gori et al., 2015a	Rotating tilted lines illusion, Accordion grating illusion	DD (only children) < in comparison to both chronological-age and reading-level Controls

## Visual Deficit in Autism Spectrum Disorder

Autism spectrum disorder (ASD) is a pervasive developmental condition characterized by abnormalities in communication, social interaction, and presence of markedly restricted interests and stereotyped behaviors (American Psychiatric Association Task Force on DSM-IV, 1994).

Although the dysfunctions in social cognition and communication are typically considered the “core” deficits in ASD individuals, a growing amount of evidence consistently reports abnormalities in low-level visual perception and attention (e.g., Happé, 1999; Dakin and Frith, 2005; Mottron et al., 2006; Simmons et al., 2009, for reviews, but see Grubb et al., 2013a,b). The idea that atypical visual processing can account for the core deficits of the disorder is one of the most intriguing aspects of the current research in autism (e.g., Vlamings et al., 2010; Mazer, 2011; Ronconi et al., 2012, 2013a,b; Manning et al., 2015).

According to the neuroconstructivist approach (see Karmiloff-Smith, 1998; Johnson, 2011, for reviews) early abnormalities in low-level perception and attention could cause the typical developmental trajectories to deviate and produce impairments in high-level cognitive domains (e.g., Mundy, 2003; Elsabbagh and Johnson, 2010; Franceschini et al., 2012; Ronconi et al., 2014b). Attentional impairments are probably the most consistently reported neurocognitive deficit in infants and children with ASD (e.g., Elsabbagh et al., 2013; see Allen and Courchesne, 2001; Ames and Fletcher-Watson, 2010; Sacrey et al., 2014, for reviews).

The attention spotlight is not only oriented in a specific location, but also has to be adjusted in its size (Eriksen and St. James, 1986; Turatto et al., 2000). This ability allows one to process visual stimuli from a narrow (zoom-in) or a broad visual region (zoom-out) (e.g., Facoetti and Molteni, 2000; Facoetti, 2001). Lovaas and Schreibman (1971) showed that children with autism responded to a restricted range of environmental stimuli, suggesting that their attention was excessively focused. The authors explained these findings in terms of “stimulus over-selectivity”. More recently, this idea was corroborated by a prolonged zoom-in and sluggish zoom-out attentional mechanism (Ronconi et al., 2012, 2013b). This abnormal attentional focusing is probably linked to dysfunctional top–down feedback from the fronto-parietal network to the early visual areas. The attentional zoom-out deficit could contribute to the atypical visual perception associated to individuals with ASD, which, in turn, could have consequences in their social-communicative development (Mann and Walker, 2003; Ronconi et al., 2012, 2013b; Robertson et al., 2013; Song et al., 2015).

Stimulus over-selectivity can also be coupled with a strong resolution for the details. Compared to typically developing peers, individuals affected by ASD manifest better performance in local element discrimination involving static visual stimuli, as in visual search tasks (e.g., O’Riordan et al., 2001; Almeida et al., 2010; Gliga et al., 2015), in the embedded figure test (e.g., Jolliffe and Baron-Cohen, 1997; Manjaly et al., 2007) and in visual crowding (Baldassi et al., 2009). An enhanced visual processing of detailed information preference in ASD was also demonstrated with electrophysiological recordings (Pei et al., 2009, 2014; Vlamings et al., 2010).

The local bias in autism seems to also occur for dynamic visual information (Chen et al., 2012). However, it is still unclear whether the perceptual bias for details (i.e., local processing) over global elements (i.e., global processing) is at the expense of the understanding of the visual scene (e.g., Chouinard et al., 2013).

## Visual Illusion in Autism Spectrum Disorder

Visual illusions are ideal for examining whether or not weak central coherence theory can explain perceptual processing in ASD (Frith and Happé, 1994; Happé and Frith, 2006; Chouinard et al., 2013). Central coherence is defined as the tendency to process incoming information in its context often with a cost regarding memory for details (Happé, 1999).

The weak central coherence theory suggests that problems with global processing in individuals with ASD can allow them to see the local elements in a very accurate way.

The weak central coherence theory has no explicit neurobiological counterpart (Caron et al., 2006). However, Milne et al. (2002) and Greenaway and Plaisted (2005) suggested that a deficit in perceiving the global aspect of visual stimuli could derive from a dysfunction of the M-D pathway. This hypothesis appears controversial and it has been criticized by several studies (Mottron et al., 2003, 2006; Bertone et al., 2005; Ronconi et al., 2012).

Based on the weak central coherence theory, ASD could be less susceptible to the visual illusions that are based on global indices or integration of local elements. During the last 15 years, several studies employed visual illusions to examine weak central coherence theory and, more in general, the perception peculiarities observed in individuals with ASD.

To our knowledge, Happé (1996) was the first to connect the world of visual illusions with the research on ASD. Her study opened a debate that is still now at the center of ASD research. Happé (1996) investigated the response to six geometrical illusions (see Ninio, 2014, for a recent review of this category of illusions) in individuals with ASD: the Ebbinghaus illusion (Ebbinghaus, 1902, also known as Titchener circles, Titchener, 1901), Ponzo Illusion (Ponzo, 1911), Müller-Lyer illusion (Müller-Lyer, 1889), Poggendorff illusion (Zöllner, 1860); Hering illusion (Hering, 1861), and the Kanizsa triangle (Kanizsa, 1955). Three groups of individuals were part of this study: a group with ASD, another one with learning difficulties and a control group of typical developing participants. The illusory patterns were standard two-dimensional (2-D) black and white line drawings, or three-dimensional (3-D) appearance patterns obtained by adding colored lines. The individuals with autism were less likely to succumb to the 2-D illusory patterns than the other groups, and were less aided by the 3-D control condition. Happé (1996) concluded that children with ASD were significantly less sensitive than control children to visual illusions. These results were interpreted in favor of weak central coherence in ASD.

All the visual illusions employed in the Happé (1996) study are clearly characterized by global information that produces illusory percepts. The Ebbinghaus illusion (Titchener, 1901; Ebbinghaus, 1902, see **Table 1** for details) produced a large number of papers (e.g., Weintraub, 1979; McCarthy et al., 2013), showing how relevant this pattern is in vision sciences.

The Ponzo illusion (Ponzo, 1911), also produced a large amount of literature (e.g., Fisher, 1968; Parks, 2013). Several studies and different interpretations were published on the Müller-Lyer illusion (Müller-Lyer, 1889, see **Table 1** for details) too (e.g., Wickelgren, 1965; de Brouwer et al., 2014). Plewan et al. (2012) using fMRI showed that the lateral occipital cortex and right superior parietal cortex were associated with illusion strength.

The Poggendorff illusion (see **Table 1** for details) was discovered by Poggendorff during the observation of the figure published by Zöllner (1860), while being the editor of the journal that first published the now very popular image. Several studies and different interpretations have since been published on this phenomenon (e.g., Green and Hoyle, 1963; Daneyko et al., 2011).

The Hering illusion (Hering, 1861, see **Table 1** for details) is also a classical illusion that has produced several studies and different interpretations (e.g., Holt-Hansen, 1973; Hamburger et al., 2007).

Finally, the Kanizsa triangle (Kanizsa, 1955) is the most famous pattern exhibiting illusory contour. The illusory contours inspired a large amount of literature (e.g., Sarti et al., 2000; Bulf et al., 2009). Neurons in area V2 and even V1 respond to the cues that induce illusory contours in human observers much in the same way as to real contours (e.g., Heitger et al., 1998; Spillmann, 2009). This is surprising as the receptive field fell in-between the inducers with no access to the surround, showing the limitation of the classical receptive field model (e.g., Peterhans and der Heydt, 1989; Soriano et al., 1996).

Hirsch et al. (1995), using fMRI, showed that specific brain regions were activated in extrastriate cortex only in presence of illusory contours. These unique regions were found primarily in the right hemisphere.

Later, Ropar and Mitchell (1999, 2001) investigated the following visual illusions in individuals with ASD: Ebbinghaus (Ebbinghaus, 1902), Ponzo (Ponzo, 1911), Müller-Lyer illusion (Müller-Lyer, 1889), and the vertical-horizontal illusion (Avery and Day, 1969). These authors – using a different control condition – did not find any significant difference in illusion perceptions between individuals with ASD and controls, arguing against the weak central coherence theory. The control stimuli were the same illusory patterns from which the global elements triggering the illusion were eliminated.

Besides the already described illusions, Ropar and Mitchell (1999, 2001) also used the vertical-horizontal illusion (Avery and Day, 1969, see **Table 1** for details).

This classical illusion is no exception in producing a large number of studies (e.g., Künnapas, 1955; Mikellidou and Thompson, 2013), but one of the most complete investigations seems to be the study by Mamassian and de Montalembert (2010), who proposed a model able to quantitatively describe the illusory effect.

Hoy et al. (2004) employed the same six visual illusions used in the study by Happé (1996). No difference in the illusory perception between individuals with ASD and typically developed controls was found.

In the same year, Rouse et al. (2004) showed no differences in the illusory perception of the Thatcher Illusion (Thompson, 1980, see **Table 1** for details) between individuals with ASD and controls. The authors concluded that children with ASD are able to compute second-order configural features in faces and they do not show differences in face processing, relative to controls.

Although not a classical illusion, the Thatcher Illusion captured a large interest in the vision sciences community, as showed by the large amount of literature that refers to it (e.g., Valentine and Bruce, 1985; Mestry et al., 2014).

Using fMRI, Psalta et al. (2014) found a peculiar involvement of the superior temporal sulcus – a region known to be linked to the facial expressions processing – only when the face of the Thatcher Illusion was upright.

Brown et al. (2005), recording with the EEG, showed gamma band abnormalities in adolescents with ASD while observing the Kanizsa triangle. However, no difference between groups in the report of the participants was observed. Simmons et al. (2009) suggested that this difference in the gamma band could be an artifact due to eye movements.

Bölte et al. (2007) employed the same six visual illusions used in the study by Happé (1996), but in a sample composed of individuals with high-functioning autism (HFA) and found that children with HFA showed a significantly less sensitivity to these visual illusions.

In the same year, Stroganova et al. (2007) found differences in ERPs between children with ASD and controls watching illusory contours (Kanizsa, 1955).

Later, Milne and Scope (2008) tested perception of illusory contours in children with ASD using a paradigm that requires participants to make a forced choice about the dimensions of a shape defined by illusory contours. There were no significant differences between the performance of children with ASD and either of the two control groups (a group of children with special educational needs and another one composed of typically developing children).

Walter et al. (2009) investigated individuals with autistic traits instead of examining susceptibility to visual illusions directly in a population with ASD, in which it is more difficult to acquire precise measurements of perception. These authors employed the Ponzo Illusion (Ponzo, 1911) and the Poggendorff illusion (Zöllner, 1860). A significant relationship was found between autistic traits and susceptibility to the tested illusions, supporting the idea that perception in autism is heavily weighted toward local features relative to typically developed individuals.

Mitchell et al. (2010) compared individuals with ASD who had IQs in the normal range against matched controls in their susceptibility to the Shepard’s (1981) illusion (see **Table 1** for details). Both groups succumbed to the illusion. However, individuals with autism were less susceptible to the illusion than typically developing controls. The authors interpreted these results as attenuated top-down influences in individuals with ASD.

Chouinard et al. (2013) also investigated individuals with autistic traits. Given that ASD is commonly associated with comorbid disorders, a psychophysical study on visual illusions in the general population is conceivably more likely to be reproducible (Chouinard et al., 2013). These authors employed the Ebbinghaus (Ebbinghaus, 1902), the Ponzo (Ponzo, 1911), and Müller-Lyer illusions (Müller-Lyer, 1889). Their findings confirm that the cognitive operations underlying global processing in the Müller-Lyer illusion are peculiar in comparison with other illusions and showed that this specific type of global processing may be affected in ASD.

In summary, visual illusions have played an important role in the study of ASD, however, in contrast to the studies involving visual illusions and DD, the results in ASD seem to be more controversial, withsome studies presenting opposite results (see following chapter for possible reasons for these outcomes). These findings should not discourage future researches from employing visual illusions to investigate atypical perception in ASD. On the contrary, it should motivate scientists to find creative solutions to reduce discrepancy. For example, the suggestion by Chouinard et al. (2013) to investigate individuals with autistic traits could be the way to follow in the future.

In sum, illusory effects driven by contextual cues (global information) were the illusions employed in almost the totality of the ASD studies reported here. This choice was the result of testing the weak central coherence hypothesis in ASD, however, this can be viewed as an opportunity for future research to employ completely different kinds of visual illusions to further investigate the peculiar way in which individuals with ASD perceptually characterize the world (see **Table 3** for a summary of reviewed studies).

**Table 3:** Overview of the published articles investigating visual illusions sensitivity in autism spectrum disorder (ASD).

Reference	Visual illusion	Main results (i.e., illusion sensitivity)
Happé, 1996	Ebbinghaus, Ponzo, Müller-Lyer, Poggendorff, Hering illusions, and Kanizsa triangle	ASD < in comparison to both Controls and children with learning difficulties
Ropar and Mitchell, 1999	Ebbinghaus, Ponzo, Müller-Lyer, and vertical-horizontal illusions	ASD = in comparison to both Controls and children with moderate learning difficulties
Ropar and Mitchell, 2001	Ebbinghaus, Ponzo, Müller-Lyer, and vertical-horizontal illusions	ASD = in comparison to both Controls and children with moderate learning difficulties
Hoy et al., 2004	Ebbinghaus, Ponzo, Müller-Lyer, Poggendorff, Hering illusions, and Kanizsa triangle	ASD = Controls
Rouse et al., 2004	Thatcher	ASD = Controls
Bölte et al., 2007	Ebbinghaus, Ponzo, Müller-Lyer, Poggendorff, Hering illusions, and Kanizsa triangle	High-functioning autism (HFA) < Controls
Stroganova et al., 2007	Illusory contours (ERP data)	ASD < Controls
Milne and Scope, 2008	Illusory contours	ASD = Controls
Walter et al., 2009	Ponzo and the Poggendorff	ASD < Controls
Mitchell et al., 2010	Shepard illusion	ASD < Controls
Chouinard et al., 2013	Ebbinghaus, the Ponzo Illusion, and the Müller-Lyer illusion.	ASD < Controls for the Müller-Lyer illusion

## Conflicting Findings About Visual Illusions in Autism Spectrum Disorder

The mixed results found for the weak central coherence hypothesis in ASD when examined by the visual illusions could be due to several factors.

The lack of significant difference observed between the ASD group and controls, such as those by Ropar and Mitchell (1999, 2001), could be due to differences in the procedure compared to Happé’s (1996) original study. Happé and Frith (2006) pointed out that Ropar and Mitchell (1999, 2001) did not used the 3-D control stimuli originally employed by Happé (1996). This control, according to Happé and Frith (2006) could be helpful in disambiguating the conflicting results.

Moreover, Happé and Frith (2006) suggested that a possible explanation for conflicting findings could be related to the information provided to the individuals with ASD. Participants with ASD made misjudgments similar to those observed in typically developed individuals when asked, for example, whether two lines of an illusion “*looked* the same length”, but were much more accurate than controls when asked whether the two lines “*were* the same length” (Happé and Frith, 2006).

Later, Walter et al. (2009) suggested that a real difference in susceptibility to visual illusions between individual with ASD and controls might have been obscured by the circumstances under which the studies were performed and the various types of control populations adopted across studies. An important difficulty is dealing with differences in mental age between the groups. Typically, selecting a mentally age matched control group for comparison with an ASD group solves this problem. However, this solution often results in a difference in chronological age between the groups. This difference could potentially be a problem while measuring illusion susceptibilities (Walter et al., 2009), since it is known that susceptibilities to visual illusion can be modulated by age (e.g., Billino et al., 2009). Some authors have suggested that visual illusion susceptibility reaches an adult-like level between the ages of 6 years (e.g., Weintraub, 1979; Zanuttini, 1996) and 15 years old (e.g., Brosvic et al., 2002; Bondarko and Semenov, 2004). On the other hand, some authors showed that this susceptibility changes throughout the life span, and it also depends from the specific pattern employed (e.g., Coren and Girgus, 1978; Billino et al., 2009). Another option could be to compare a group of individuals with ASD to a control group matched for chronological age, but this would introduce a confound, if illusion susceptibility is instead correlated to mental age (Walter et al., 2009).

Another potential source of conflicting findings can be the heterogeneity of the ASD group itself. This would be particularly problematic when the ASD group contains individuals across the autistic spectrum (Walter et al., 2009). Moreover, even in the presence of similar diagnoses, the heterogeneity in cognitive abilities and severity of impairments recorded among individuals with ASD often remains great (Baron-Cohen et al., 2003; Belmonte et al., 2004). The individual differences in the ASD group are all the more magnified when comorbid disorders are present (Billstedt, 2000). Considering that the samples of individuals with ASD in the above reported studies are always relatively small, this within-group heterogeneity could sometimes potentially obscure a real difference between groups (Walter et al., 2009).

Also Chouinard et al. (2013), highlighted the heterogeneous nature of ASD (Billstedt, 2000) as a possible cause of conflicting results in studies investigating visual illusions in this clinical population.

Moreover, Chouinard et al. (2013) stressed that there are a several difficulties in carrying out well-controlled visual psychophysical experiments in individuals with ASD, such as variability in compliance, instruction comprehension, and perseverative behaviors.

In addition, the sluggish zoom-out of attentional focus showed in individuals with ASD (Mann and Walker, 2003; Ronconi et al., 2012, 2013b; Robertson et al., 2013; Song et al., 2015) could be another source of conflicting results. Different sizes and durations of the illusory stimuli might change results radically due to the difficulty in zooming out characterized in individuals with ASD.

Both, Walter et al. (2009) and Chouinard et al. (2013) proposed comparing measures of autistic tendencies to individual differences in visuospatial processing ability within a typically developing population.

Simmons et al. (2009) agreed that this methodology can be an alternative way to study the impact of autistic traits on visuospatial processing, without introducing confounds like age differences, comorbidity, and variable symptoms. The use of continuous measures instead of dichotic classification (“ASD” or “typical”) as a means of quantifying particular autistic traits, allows correlations between performance on visuospatial tasks and trait strengths to be measured (Walter et al., 2009).

Moreover, it is possible to study the effects of a single autistic trait rather than treating all traits associated with ASD as a whole (Walter et al., 2009; Ronconi et al., 2014b). An additional advantage of this continuous-trait approach is the possibility of easily recruiting much larger samples; this option allows for much greater data collection than when limiting recruitment those diagnosed with ASD. Interestingly, both studies employing this methodology (Walter et al., 2009; Chouinard et al., 2013) reported that high autistic traits are related to less susceptibility to some visual illusions.

However, as noticed by Chouinard et al. (2013), the extent to which these results can generalize to the individuals with ASD may need further investigation.

In summary, the presence of findings that seem to be sometimes conflicting when the susceptibility of visual illusion is measured in groups of people with ASD can be the result of several factors. Different procedures employed, variations in the instructions provided to the participants, differences in control groups and sample sizes, and last but not least, the heterogeneous nature of ASD itself, all seem to be factors that can lead to different results. It is worth noting that, probably because of some aforementioned factors, research in ASD often provides conflicting results. For example, there is no consensus regarding the brain areas involved in the disorder or in its genetic bases (Amaral et al., 2008).

## Conclusion: New Perspectives on the Involvement of Visual Illusions in the Study of Developmental Dyslexia and Autism Spectrum Disorder

The contribution of visual illusions to our understanding of neurodevelopmental disorders seems far from reaching its peak. After reviewing the studies that employed visual illusions to unveil brain mechanisms in DD and ASD, it appears that there have been important steps forward. One aspect that could be improved upon is the method by which visual illusion sensitivity is measured. While simply asking if the illusion is perceived or not has the benefit of making the task easier for participants, which are often children or sometimes adult with communications difficulties (i.e., some individuals with ASD), it is probably not the most accurate approach. Constant stimuli or staircases with nulling paradigms (in which different levels of physically countering information are presented to the observer until the illusory effect is nullified) could be the proper approach for obtaining a much more precise measure of visual illusion sensitivity in future research. The challenge will be to develop a paradigm with these methodologies that is still simple enough to be performed by children and individuals with difficulties in communication.

Moreover, we also suggest utilizing visual illusions for other interesting challenges such as:

(i) *The early identification of the disorders.* DD diagnosis, for example, can be done, by definition, only after reading acquisition. It implies that any potential treatment cannot start before the child reaches 7 or even 8 years old. On the contrary, a deficit in the M-D pathway can be identified much sooner, potentially at infancy. Consequently, developing new visual illusion tasks, capable of testing M-D functionality, can lead to earlier identification of children at risk for DD. Early identification will have a positive cascading effect in the battle against DD and its negative outcomes. Children identified as at risk for DD could be specifically treated well before the typical age of diagnosis. This preventative approach would improve existing cognitive deficits, and children would potentially reach the age of reading acquisition with a much better toolbox at their disposal for facing the challenge of learning to read. Moreover, brain plasticity is much more evident in the early years of life, and treatment would have a better chance of being effective. Prevention is undeniably the main goal in DD research, and visual illusions could play a leading role in achieving this goal.
Regarding ASD, diagnosis can be done in children as young as 2 years old. However, developing paradigms for infants to show a reduced sensitivity to visual illusions could make the identification of risk for ASD even earlier. Moreover, it will provide other tools to use together with the more traditional measurements in order to make the diagnosis more reliable.
(ii) *Longitudinal studies.* Another connected area of research where visual illusions could play an important role is in longitudinal studies. These studies are crucial to proving the existence of a causal link between a specific cognitive deficit and the neurodevelopmental disorder. Visual illusions, measuring the M-D functionality, could be employed at the pre-reading stage. The aim would be discover if future reading disabilities could be explained by a pre-existing deficit in the M-D pathway, and thus eliminate any confound related to noise exclusion mechanisms.
Also for ASD, a longitudinal study showing that less sensitivity to illusions in infancy is causally linked to future ASD symptoms would be of utmost importance, from both theoretical and practical reasons. Such a result result would prove that the weak central coherence is a cause and not a consequence of ASD, and early treatments to strengthen the central coherence in infants at risk for ASD could be developed.
(iii) *Efficacy of treatment tests.* Finally, visual illusions could be employed to test the efficacy of a treatment for DD or ASD. It is expected that an effective treatment would show a correlation between the reduction of neurodevelopmental disorder symptoms and performance on the visual illusions task.

This role of efficacy tests for treatments could also be extended to prevention programs aiming to reduce the incidence of DD or ASD.

## Conclusion

The use of visual illusions as a tool for investigating the brain mechanisms of individuals with neurodevelopmental disorders has already shown to be a very successful approach, and it has the possibility to offer answers in future exciting challenges.

## Author Contributions

All authors listed, have made substantial, direct and intellectual contribution to the work, and approved it for publication.

## Conflict of Interest Statement

The authors declare that the research was conducted in the absence of any commercial or financial relationships that could be construed as a potential conflict of interest.
